# Preclinical development of HQP1351, a multikinase inhibitor targeting a broad spectrum of mutant KIT kinases, for the treatment of imatinib-resistant gastrointestinal stromal tumors

**DOI:** 10.1186/s13578-019-0351-6

**Published:** 2019-10-26

**Authors:** Xuechao Liu, Guangfeng Wang, Xianglei Yan, Haibo Qiu, Ping Min, Miaoyi Wu, Chunyang Tang, Fei Zhang, Qiuqiong Tang, Saijie Zhu, Miaozhen Qiu, Wei Zhuang, Douglas D. Fang, Zhiwei Zhou, Dajun Yang, Yifan Zhai

**Affiliations:** 1 Department of Gastric Surgery, State Key Laboratory of Oncology in South China, Collaborative Innovation Center for Cancer Medicine, Sun Yat-Sen University Cancer Center, Guangzhou, 510060 People’s Republic of China; 2 Ascentage Pharma (Suzhou) Co., Ltd., 218 Xinghu Street, Suzhou Industrial Park, Suzhou, 215100 People’s Republic of China; 3 Department of Experimental Research, State Key Laboratory of Oncology in South China, Collaborative Innovation Center for Cancer Medicine, Sun Yat-Sen University Cancer Center, Guangzhou, 510060 People’s Republic of China; 4 Department of Medical Oncology, State Key Laboratory of Oncology in South China, Collaborative Innovation Center for Cancer Medicine, Sun Yat-Sen University Cancer Center, Guangzhou, 510060 People’s Republic of China; 5 HealthQuest Pharma Inc., Room 314, Building F, 3 Lanyue Road, Science City, Huangpu, Guangzhou, 510663 People’s Republic of China; 6 Department of General Surgery, Affiliated Hospital of Qingdao University, 16# Jiangsu Road, Qingdao, Shandong People’s Republic of China

**Keywords:** Gastrointestinal stromal tumor, Drug resistance, Kit tyrosine kinase, Imatinib

## Abstract

**Background:**

Imatinib shows limited efficacy in patients with gastrointestinal stromal tumors (GISTs) carrying secondary KIT mutations. HQP1351, an orally bioavailable multikinase BCR-ABL inhibitor, is currently in clinical trials for the treatment of T315I mutant chronic myelogenous leukemia (CML), but the potential application in imatinib-resistant GISTs carrying secondary KIT mutations has not been explored.

**Methods:**

The binding activities of HQP1351 with native or mutant KIT were first analyzed. Imatinib-sensitive GIST T1 and imatinib-resistant GIST 430 cells were employed to test the in vitro antiproliferative activity. Colony formation assay, cell migration assay and cell invasion assay were performed to evaluate the clonogenic, migration and invasion ability respectively. Flow cytometry and western blot analysis were used to detect cell apoptosis, cell cycle and signaling pathway. In vivo antitumor activity was evaluated in mouse xenograft models derived from GIST cell lines.

**Results:**

HQP1351 potently inhibited both wild-type and mutant KIT kinases. In both imatinib-resistant and sensitive GIST cell lines, HQP1351 exhibited more potent or equivalent antiproliferative activity compared with ponatinib, a third generation BCR-ABL and KIT inhibitor. HQP1351 led to more profound inhibition of cell colony formation, cell migration and invasion, cell cycle arrest and cell apoptosis than ponatinib. Furthermore, HQP1351 also inhibited p-KIT, p-AKT, p-ERK1/2, and p-STAT3 to a higher extent than ponatinib. Finally, in xenograft tumor models derived from imatinib-resistant GIST cancer cell lines, HQP1351 exhibited antitumor activity superior to ponatinib.

**Conclusions:**

Collectively, our in vitro and in vivo results suggest that the therapeutic application of HQP1351 in imatinib-resistant GIST patients deserves further investigation in clinical trials.

## Introduction

Gastrointestinal stromal tumors (GISTs) are the most common gastrointestinal mesenchymal tumors that often develop drug resistance during routine first-line chemotherapy and radiotherapy [1]. Activation of KIT tyrosine kinase [2] or platelet-derived growth factor receptor (PDGFR) [3–5] is the main cause of GISTs. Gain-of-function mutation at *KIT* is found in around 75% of GISTs [6, 7]. These primary activation mutations are usually located in *KIT* exons 9 and 11. Imatinib (Gleevec), a small molecule inhibitor against BCR-ABL, KIT, and PDGFR, shows the inhibitory effect on GISTs and has been approved for KIT-positive GIST as the first-line treatment. However, its efficacy is significantly reduced by secondary mutations of *KIT* [8], which are mostly located in the ATP-binding pocket (exons 13 and 14) and the activation loop (A-loop, exons 17 and 18) [9]. In addition to imatinib, sunitinib (Sutent) and regorafenib (Stivarga) have also been approved for GISTs. Sunitinib inhibits VEGFR2 (Flk-1) and PDGFRβ, as well as KIT activity. It is used to treat imatinib-resistant GIST patients, as it is able to inhibit KIT mutation in the ATP binding pocket [10]. Regorafenib inhibits a series of primary and secondary KIT mutants, especially KIT isoforms with A-loop secondary mutations [11]. Clinical trials have demonstrated its benefit in GIST patients harboring secondary mutations of exon 17 [12]. However, regorafenib is significantly less effective than sunitinib in KIT V654A secondary mutation [11], i.e. ATP-binding mutation.

Ponatinib (AP24534) is a BCR-ABL (T315I) inhibitor belonging to the third generation. It was approved in 2012 to treat Philadelphia chromosome-positive (Ph+) acute lymphoblastic leukemia (ALL) [9] and chronic myeloid leukemia (CML) patients with resistance or intolerance to prior TKI therapy [13, 14]. Ponatinib also inhibits FGFR [15] and FLT3 [16]. In addition, ponatinib has inhibitory effects on different KIT mutants, including mutations in the ATP-binding pocket and the A-loop [11]. The inhibitory profile of ponatinib was similar to regorafenib, but its potency was much stronger than regorafenib in all KIT mutants [11]. It has been shown that, in clinical trials, ponatinib at a dose of 30 mg daily is effective in 2 out of 3 GIST patients, suggesting that ponatinib may be used as a KIT inhibitor in the treatment of resistant GISTs. In a recent phase II clinical trial, ponatinib showed clinical activity in advanced GIST patients after the failure of the treatment with TKI therapies, particularly in the patients with *KIT* exon 11 mutations [17]. While ponatinib showed the promise in treating drug resistant GISTs, the risk of life-threatening cardiovascular side effects limited its clinical application [18].

Acquired and developed by Ascentage Pharma, HQP1351 is a third-generation BCR-ABL inhibitor [19]. Based on the preliminary results of Phase I study, HQP1351 has demonstrated clinical efficacy in CML resistant to current TKI-therapies including those with T315I mutation with manageable side effects [20]. In the current study, through preclinical models, we investigated the ability of HQP1351 to overcome drug resistance for the treatment of GISTs.

## Materials and methods

### Cell lines

Gastrointestinal stromal cells (including GIST T1 and GIST 430) carrying KIT mutations were a gift from Professor Haibo Qiu (Sun Yat-Sen University Cancer Center, Guangdong, China) and cultured as described in previous literatures [21, 22]. Neither cell line matched with any reference cell line in the cell bank database (ATCC, DSMZ, JCRB and RIKEN), as indicated by less than 80% of match in STR profiles (data not shown). Their KIT mutations were validated by PCR and sequencing (data not shown) and were shown in the Table 2. GIST T1 cell line was cultured in RPMI 1640 medium (GIBCO, Grand Island, NY, USA) containing 300 mg/L l-glutamine and 2.0 g/L sodium bicarbonate, which was supplemented with 15% fetal bovine serum, 1% Penicillin/Streptomycin, 1% l-glutamine and 2 μg/mL Gentamycin. GIST 430 cell line was cultured in a 50:50 (v/v) mixture of DMEM/Ham’s F-12 supplemented with 15% fetal bovine serum, 1% Penicillin/Streptomycin, 1% l-glutamine and 2 μg/mL Gentamycin. In addition, 200 nM imatinib was added into GIST 430 cells to maintain drug resistance. These two cell lines were maintained at 37 °C in a humidified atmosphere of 5% CO_2_. All the reagents used for cell culture were from Shanghai Basalmedia Technologies Co., Ltd. (Shanghai, China), unless specified elsewhere.

### Compounds

HQP1351 was provided by Ascentage Pharma Group Corp., Ltd. (Jiangsu, China). Imatinib was purchased from Aikang Biopharmaceutical Co., Ltd. (Jiangsu, China). Ponatinib was purchased from Ailikaide Chemical Engineering Co., Ltd. (Jiangsu, China). Regorafenib and sunitinib were purchased from Selleck (Shanghai, China).

### Kinase binding assays

The binding activities of HQP1351 at the concentration of 10 and 100 nM with native or mutant KIT were analyzed by KINOMEscan™ kinase assay system. Briefly, when the competition reaction was completed, the amount of kinase captured by the immobilized ligand was quantified with quantitative PCR. Inhibition rate (%) of HQP1351 against native or mutant KIT was then calculated to evaluate the inhibition potency. Data was represented as a percentage of control (% Ctrl) where 100% indicated that the test compound did not inhibit kinase activity and lower numbers indicated stronger hits. Negative control was DMSO control (100% Ctrl), and positive control was control compound (0% Ctrl). Inhibition rate (%) was calculated by subtracting % Ctrl from 100.

### In vitro antiproliferative assays

The anti-proliferative activity was determined by a water-soluble tetrazolium (WST)-based assay using Cell Counting Kit-8 (CCK-8) (Tianjin Biolite Biotech Co., Ltd., Tianjin, China). Briefly, GIST cells were treated with a series of concentrations of test articles for 72 h. Each treatment was tested in 3 replicates. At the end of the treatments, CCK-8 reagent (Tianjin Biolite Biotech Co., Ltd., Tianjin, China) was added for another 2–4 h of incubation. A microplate reader was used to determine the OD450 value of each sample. The cell viability was calculated using the mean OD value of replicated wells using the following equation: (OD sample − OD blank)/(OD cell control − OD blank) × 100. GraphPad Prism software (version 6.0, Golden software, CA, USA) was employed to calculate IC_50_ values of each drug. The experiment was repeated twice, and IC_50_ was expressed as mean ± standard deviation (SD).

### Colony formation assay

GIST 430 cells were incubated with 0.3 μM imatinib, ponatinib or HQP1351 for 14 days at the density of 1000 cells/well. After the removal of drug-containing medium, the cells were fixed, followed by the staining with 0.5% crystal violet. Finally, the colonies were photographed, and the colony numbers were counted using AlphaImager HP system (Proteinsimple, CA, USA).

### Cell migration assay

GIST 430 cells were seeded at the density of 10^6^ cells/well. A P-1000 pipette was used to create a slash in the near-confluent cell cultures, which was then further incubated with 0.3 μM imatinib, ponatinib or HQP1351. The average extent of wound closure was evaluated on day 0, 3, and 5 under an inverted microscope (Leica Microsystems, Germany). Each treatment was tested in 3 replicates.

### Cell invasion assay

Transwell invasion assay was conducted using 24-well Transwell chamber with an 8 μm pore size polycarbonate filter membrane (Corning, NY, USA). Approximately 5 × 10^4^ GIST 430 cells in 200 μL FBS-free culture media were seeded onto the upper chamber, and 600 μL culture media with 10% FBS were added in the lower chamber. After further incubation with 0.3 μM imatinib, ponatinib or HQP1351 for 24 h, the cells on the upper side of the wells were softly scraped off. Cells that migrated to the lower side of the wells were fixed by methanol and stained with 0.1% crystal violet for 30 min, then photographed by microscope. Each treatment was tested in 3 replicates.

### Cell cycle analysis

GIST 430 cells were first incubated with imatinib, ponatinib or HQP1351 at 0.3 μM for 24 h. Then, the cells were washed with PBS, trypsinized and harvested by centrifugation. The obtained cell pellet was fixed with cold ethanol (70%) at 4 °C for 4 h. Finally, a FACS Calibur flow cytometer (BD Biosciences, CA, USA) was used to analyze the cell cycle distribution right after the nuclear staining with propidium iodide (PI).

### Apoptosis analysis

GIST 430 cells were first treated with imatinib, ponatinib or HQP1351 at 0.3 μM for 48 h. Then, the cells were washed with PBS, trypsinized and harvested by centrifugation. Finally, the obtained cell pellet was stained with Annexin-V-fluorescein isothiocyanate (FITC)/PI, and subjected to a FACS flow cytometer (BD Biosciences, CA, USA). A minimum of 30,000 events were analyzed.

### Western blot analysis

GIST T1 and GIST 430 cells were treated with the indicated concentrations of HQP1351, ponatinib, or imatinib for 24 and 72 h, and dissolved in 1 × SDS sample lysis buffer. The protein lysates after sonication and boiling were separated by electrophoresis using 10% SDS-PAGE and transferred to a PVDF membrane. After being blocked with 1 × TBS containing 0.1% Tween-20 and 5% nonfat milk, the membrane was then incubated with the primary antibodies followed by HRP-conjugated secondary antibody [Goat Anti-Rabbit IgG (H + L), MultiSciences (Lianke) Biotech Co., Ltd., Hangzhou, China]. Finally, bound secondary antibody was visualized using ECL Western Blotting Detection Kit (Yeasen Biotech Co., Ltd., Shanghai, China). The primary antibody include c-KIT (D13A2) XP^®^ rabbit monoclonal antibody (mAb), phospho-c-KIT (Tyr703) (D12E12) rabbit mAb, SRC (36D10) rabbit mAb, phospho-SRC family (Tyr416) (D49G4) rabbit mAb, AKT (pan) (11E7) rabbit mAb, phospho-AKT (Ser473) (D9E) XP^®^ rabbit mAb, p44/42 MAPK (ERK1/2) (137F5) rabbit mAb, phospho-p44/42 MAPK (ERK1/2) (Thr202/Tyr204) (197G2) rabbit mAb, STAT3 (124H6) mouse mAb and phospho-STAT3 (Tyr705) antibody. All of the primary antibodies were purchased from Cell Signaling Technology (MA, USA). Anti-β-Actin antibody mouse mAb was from Sigma-Aldrich (St. Louis, MO, USA).

### In vivo efficacy study

All animal experiments were approved by Shanghai Laboratory Animal Research Center (Shanghai, China) and Shanghai Ascentage Pharmaceutical Technology Co., Ltd. (Shanghai, China). Tumors were established by subcutaneous implantation of GIST T1 or GIST 430 cells (5–10 × 10^6^/0.2 mL) into the right flank of each mouse (female, 6–8 weeks old). Balb/c nu/nu mice (Shanghai Laboratory Animal Research Center, Shanghai, China) and SCID Beige mice (Vital River Laboratory Animal Technology Co., Ltd., Beijing, China) were used for the implantation of GIST T1 cells and GIST 430 cells, respectively.

In efficacy studies, mice were randomly assigned to different groups when the average tumor volume reached 50–200 mm^3^. Mice were then treated by oral gavage with HQP1351, ponatinib, or vehicle following predefined dosing regimens. Both HQP1351 and ponatinib were administered as a suspension in 0.2% HPMC (Colorocon, Shanghai, China) in water. At a predefined time point, the tumor size was measured, and tumor volume was calculated (width^2^ × length/2). The body weight of mice was recorded as well. At the end of experiments, T/C% and RTV (relative tumor volume) were calculated to evaluate the antitumor activity of the test articles. T and C were the mean volume of the tumors from the treatment groups and control group respectively. The tumor volume at the end of experiment was expressed as RTV and calculated by dividing the absolute tumor volume at the end of experiment with the absolute tumor volume at day 1.

### Data analysis

In vitro data were expressed as mean ± SD. Tumor volume and body weight data were expressed as mean ± SEM. Statistical analyses were performed using one-way ANOVA with post hoc Tukey’s HSD test. Statistical significance was accepted at the level of *P* < 0.05. All data in the study have been recorded at Sun Yat-sen University Cancer Center for future reference (number RDDB2018000420).

## Results

### HQP1351 inhibits both wild-type and various mutant KIT kinases

The inhibitory effect of HQP1351 on various KIT kinases was assessed in biochemical assay. As shown in Table 1, at the concentration of 10 nM, HQP1351 strongly inhibited wild-type KIT kinase and KIT kinases with primary mutations within exon 11 (L576P and V559D), with an inhibition rate of over 90%. Specifically, HQP1351 showed strong inhibitory effect on KIT kinase with secondary mutations in the ATP-binding pocket (V559D/T670I and V559D/V654A). The inhibition rate on KIT with V559D/T670I double mutations was > 90% at 10 nM. The inhibition rate on KIT with V559D/V654A double mutations was around 40% at 10 nM, and further increased to > 90% at 100 nM. The inhibition rates on KIT with A-loop mutations (A829P, D816H and D816 V) were relatively weaker at 10 nM (20–40%) and increased to 50–80% at 100 nM. To assess the selectivity of HQP1351, KINOMEscan™ screening assay with 442 kinases was performed. The results revealed that HQP1351 had potent binding affinities to additional kinases at 10 nM, including BRAF (V600E), DDR1, FLT3, PDGFRB, RET (M918T), TAK1 and TIE2 (Additional file 1: Table S1). The data suggest that HQP1351 is a multi-target kinase inhibitor.

**Table 1:** Inhibition of KIT kinases by HQP1351 at two different concentrations

*KIT* mutation	KIT exon	% of control		Inhibition rate (%)	
		10 nM	100 nM	10 nM	100 nM
*KIT (wild*-*type)*	–	2.8	0.05	97.2	99.95
*KIT* (L576P)	11	2.2	0	97.8	100
*KIT* (V559D)	11	1.4	0.05	98.6	99.95
*KIT* (A829P)	18	56	48	44	52
*KIT* (D816H)	17	58	28	42	72
*KIT* (D816 V)	17	83	21	17	79
*KIT* (V559D, T670I)	11/14	8.1	0.55	91.9	99.45
*KIT* (V559D, V654A)	11/13	56	6.2	44	93.8

### HQP1351 shows antiproliferative activity in GIST cells with KIT mutations

The antiproliferative activity of HQP1351 on GIST cells harboring primary and secondary KIT mutations was then investigated. Two GIST cell lines, which have been described previously [21, 22], were used in this study (Table 2). Briefly, HQP1351 showed potent inhibitory effect on the growth of GIST T1 cells harboring primary KIT mutations within exon 11, with IC_50_ values similar with ponatinib, sunitinib, regorafenib and imatinib. The IC_50_ values for HQP1351, ponatinib, sunitinib, regorafenib and imatinib in GIST T1 cells were 0.027 ± 0.021 μM, 0.021 ± 0.000 μM, 0.015 ± 0.012 μM, 0.079 ± 0.046 μM and 0.027 ± 0.005 μM, respectively. Essentially, GIST T1 cells are sensitive to all the three generations of inhibitors tested, including imatinib, sunitinib, regorafenib, ponatinib and HQP1351.

**Table 2:** Proliferation inhibitory effect of HQP1351 on GIST cells

Cell	KIT genotype	Sensitivity to imatinib	IC_50_ (μM)				
			HQP1351	Ponatinib	Sunitinib	Regorafenib	Imatinib
GIST T1	exon 11, △560–578	Sensitive	0.027 ± 0.021	0.021 ± 0.000	0.015 ± 0.012	0.079 ± 0.046	0.027 ± 0.005
GIST 430	exon 11, △560–576	Resistant	0.091 ± 0.021	0.134 ± 0.057	0.485 ± 0.168	3.321 ± 1.261	1.620 ± 0.318
	exon13, V654A						

The antiproliferative activity of imatinib was significantly reduced (> tenfold) in GIST 430 cells which carried the secondary mutation in ATP-binding pocket, implicating that the imatinib-resistance was driven by this mutation. The IC_50_ values of imatinib in this cell line was 1.620 ± 0.318 μM. Similarly, sunitinib and regorafenib also showed around tenfold decrease of antiproliferative activity in this cell line. In contrast, HQP1351 was more effective in this imatinib-resistant GIST cell line. The IC_50_ value of HQP1351 in GIST 430 cells was 0.091 ± 0.021 μM. The potency of HQP1351 on this mutant is slightly stronger than ponatinib whose IC_50_ values was 0.134 ± 0.057 μM in GIST 430 cells (Table 2).

Taken together, HQP1351 exhibits antiproliferative activity in KIT mutant GIST cells and potentially overcomes the drug resistance conferred by the secondary mutations in ATP-binding pocket. The antiproliferative activity of HQP1351 is slightly more potent than another third generation TKI ponatinib in imatinib-resistant GIST 430 cells. These results indicate that HQP1351 may be effective in the treatment of GIST patients resistant to ponatinib and imatinib.

### HQP1351 inhibits colony formation, cell migration and invasion

**Fig. 1:**
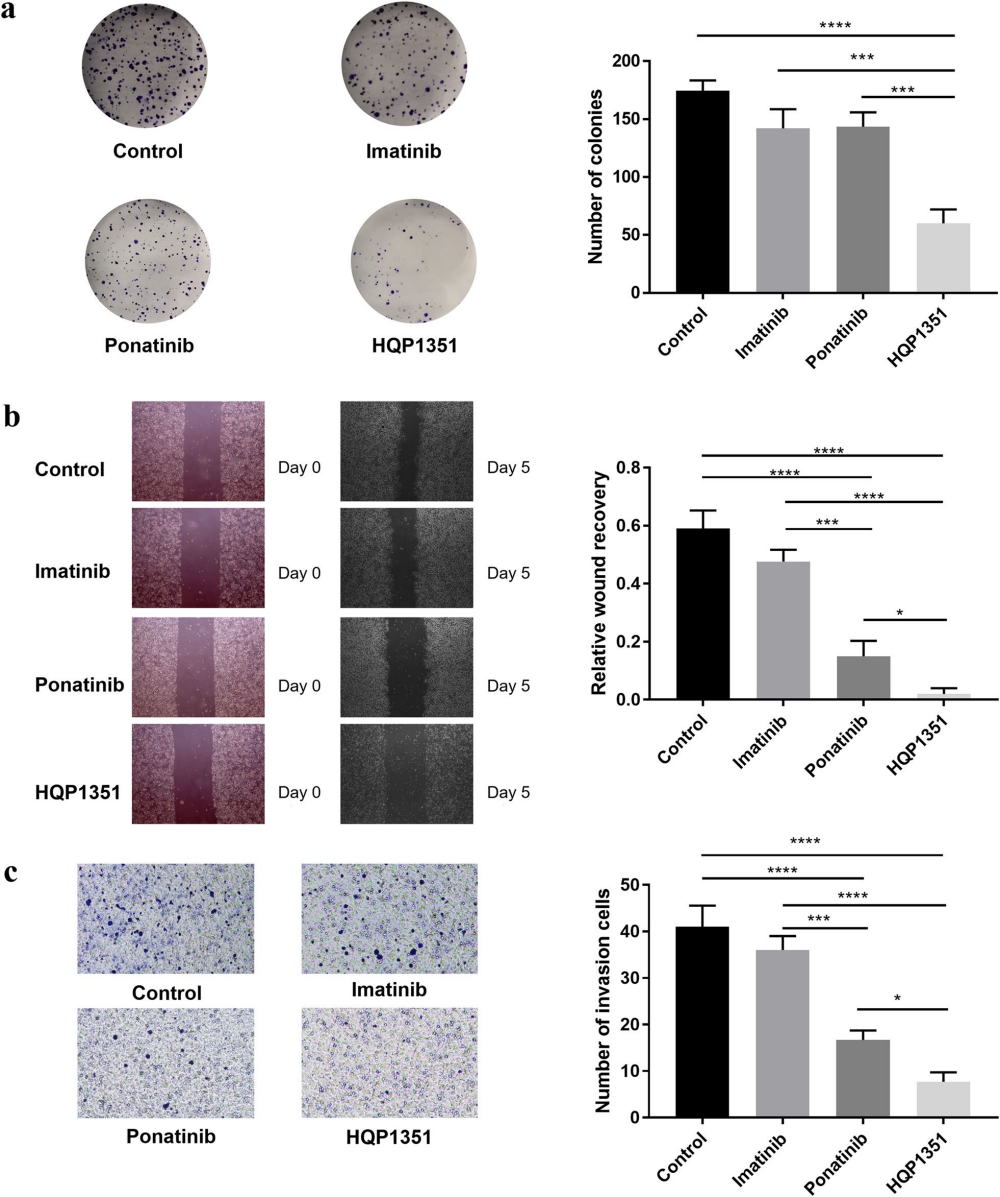
HQP1351 inhibits colony formation, cell migration and invasion. **a** GIST 430 cells were incubated with 0.3 μM imatinib, ponatinib or HQP1351 for 14 days, and then stained with 0.5% crystal violet to count the number of cell colony. **b** Near-confluent GIST 430 cells were slashed and further incubated with 0.3 μM imatinib, ponatinib or HQP1351 for 5 days. The average extent of wound closure was evaluated on day 5. **c** GIST 430 cells were seeded on the upper chamber of a Transwell plate and incubated with 0.3 μM imatinib, ponatinib or HQP1351 for 24 h. The cells that migrated to the lower side of the wells were stained with 0.1% crystal violet and counted. *P < 0.05, ***P < 0.001, ****P < 0.0001, n = 3

A colony formation assay was also performed to further evaluate the antiproliferative activity of HQP1351 on imatinib-resistant GIST 430 cells. As shown in Fig. 1a, treatment of GIST 430 cells with 0.3 μM imatinib for 14 days only resulted in slightly less number and smaller size of colonies than the control (*P* > 0.05). Similar level of inhibitory effect was observed for ponatinib (*P* > 0.05 vs. control). On the other hand, HQP1351 showed significantly stronger inhibitory effect on colony formation as compared with ponatinib, imatinib and control (*P* < 0.0001).

To determine the effect of HQP1351 on cell migration ability, we performed wound healing assays in GIST 430 cell line. As seen in Fig. 1b, imatinib (0.3 μM) showed slight inhibitory effect on the migration of GIST 430 cells after 5 days of treatment as compared with the control (*P* > 0.05). On the other hand, ponatinib and HQP1351 significantly inhibited the migration of GIST 430 cells by impairing wound closure under the same condition (*P* < 0.0001 vs. control), with HQP1351 showing an even stronger effect (*P* < 0.05 vs. control).

In order to rule out the possibility that the impaired wound closure was caused by HQP1351-mediated cell growth inhibition, a Transwell invasion assay was performed to further evaluate the effect of HQP1351 on cell invasion. As shown in Fig. 1c, imatinib (0.3 μM) only slightly reduced the number of GIST 430 cells passed through membrane compared with the control (*P* > 0.05). Ponatinib and HQP1351 significantly inhibited the invasion capability of GIST 430 cells by reducing the cell number passed through the membrane (*P* < 0.0001 vs. control). Of note, compared with ponatinib, HQP1351 showed more potent inhibitory effect on cancer cell invasion (*P* < 0.05).

### HQP1351 induces cell cycle arrest and cell apoptosis

**Fig. 2:**
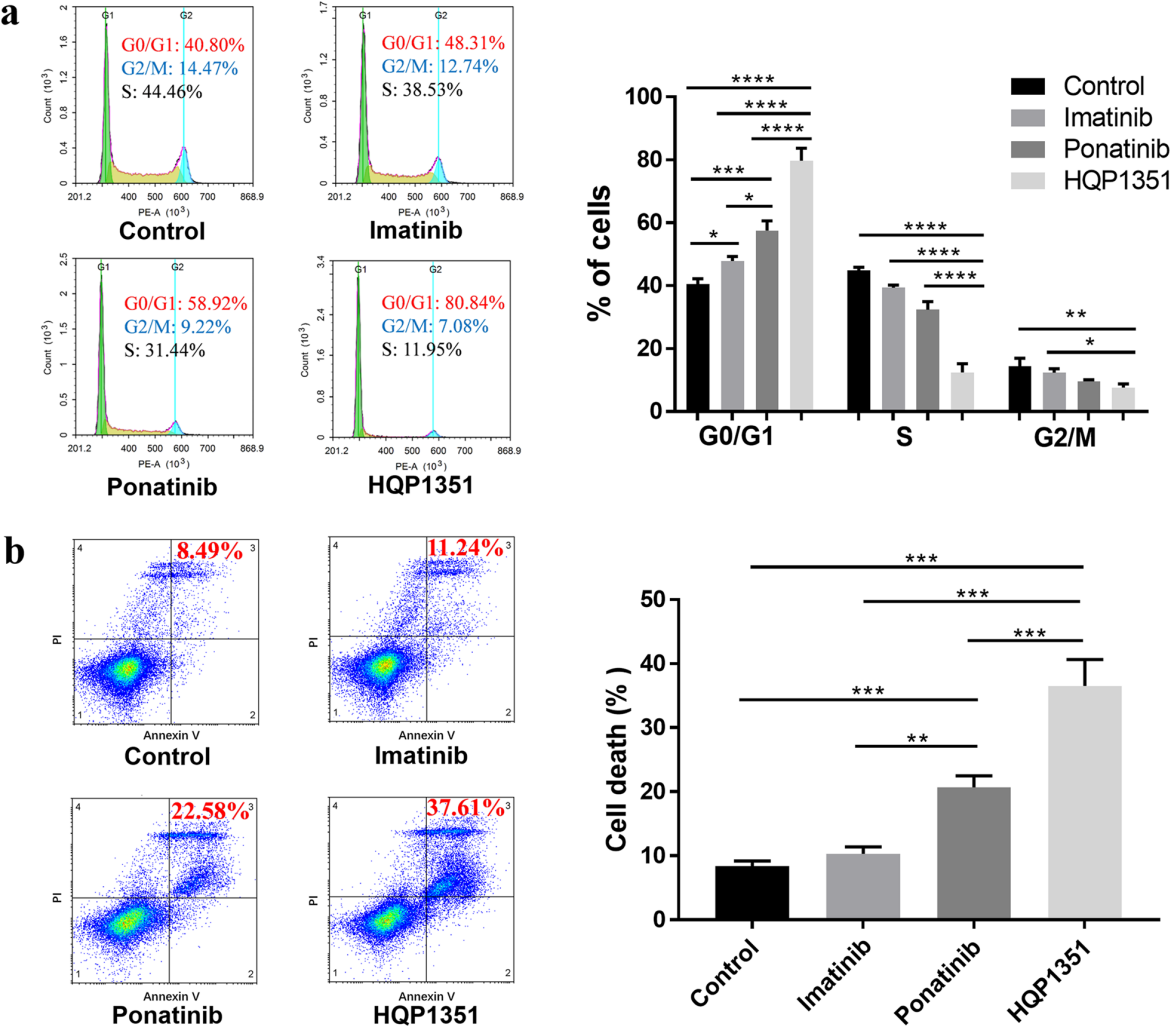
HQP1351 induces cell cycle arrest and apoptosis. **a** GIST 430 cells were treated with vehicle, imatinib, ponatinib or HQP1351 at 0.3 μM for 24 h, and then stained with PI for cell cycle analysis. **b** GIST 430 cells were treated with vehicle, imatinib, ponatinib or HQP1351 at 0.3 μM for 48 h, and then stained with Annexin V-FITC and PI for apoptosis analysis. *P < 0.05, **P < 0.01, ***P < 0.001, ****P < 0.0001, n = 3

In order to understand the mechanism of cell growth inhibition by HQP1351, cell cycle analysis was performed to determine whether it induced cell cycle arrest at specific stages. As seen in Fig. 2a, treatment of GIST 430 cells with imatinib (0.3 μM, 24 h) increased the proportions of the G0/G1 fraction from 40.50 ± 1.67% to 47.90 ± 1.35%, as compared with control (*P* < 0.05). Correspondingly, the proportions of S and G2/M fractions of GIST 430 cells decreased after imatinib treatment as compared with control. The proportion of the G0/G1 fraction of GIST 430 cells further increased to 57.54 ± 3.09% (*P* < 0.001 vs. control) and 79.71 ± 3.97% (*P* < 0.0001 vs. control) when treated with ponatinib and HQP1351 at the same concentration and duration, indicating their stronger ability to induce cell cycle arrest. Of note, the ability of HQP1351 to induce cell cycle arrest, as indicated by the increased proportion of the G0/G1 fraction, was 1.66 and 1.39 times that of imatinib and ponatinib respectively (*P* < 0.0001), suggesting its superiority over these two drugs in preventing cell growth.

Cell apoptosis was evaluated using flow cytometry by Annexin V-FITC/PI labeling. As seen in Fig. 2b, treatment of GIST 430 cells with imatinib (0.3 μM, 48 h) slightly increased the proportion of late apoptotic cells from 8.36 ± 0.81% to 10.31 ± 1.10%, as compared with control (*P* > 0.05). The proportion of late apoptotic GIST 430 cells further increased to 20.69 ± 1.80% (*P* < 0.001 vs. control) and 36.54 ± 4.11% (*P* < 0.001 vs. control) when treated with ponatinib and HQP1351 at the same concentration and duration, indicating their stronger ability to induce cell apoptosis. Notably, the ability of HQP1351 to induce cell apoptosis was 3.54 and 1.77 times that of imatinib and ponatinib respectively (*P* < 0.001), suggesting its superiority over these two drugs.

### HQP1351 regulates KIT oncogenic signaling proteins in vitro

**Fig. 3:**
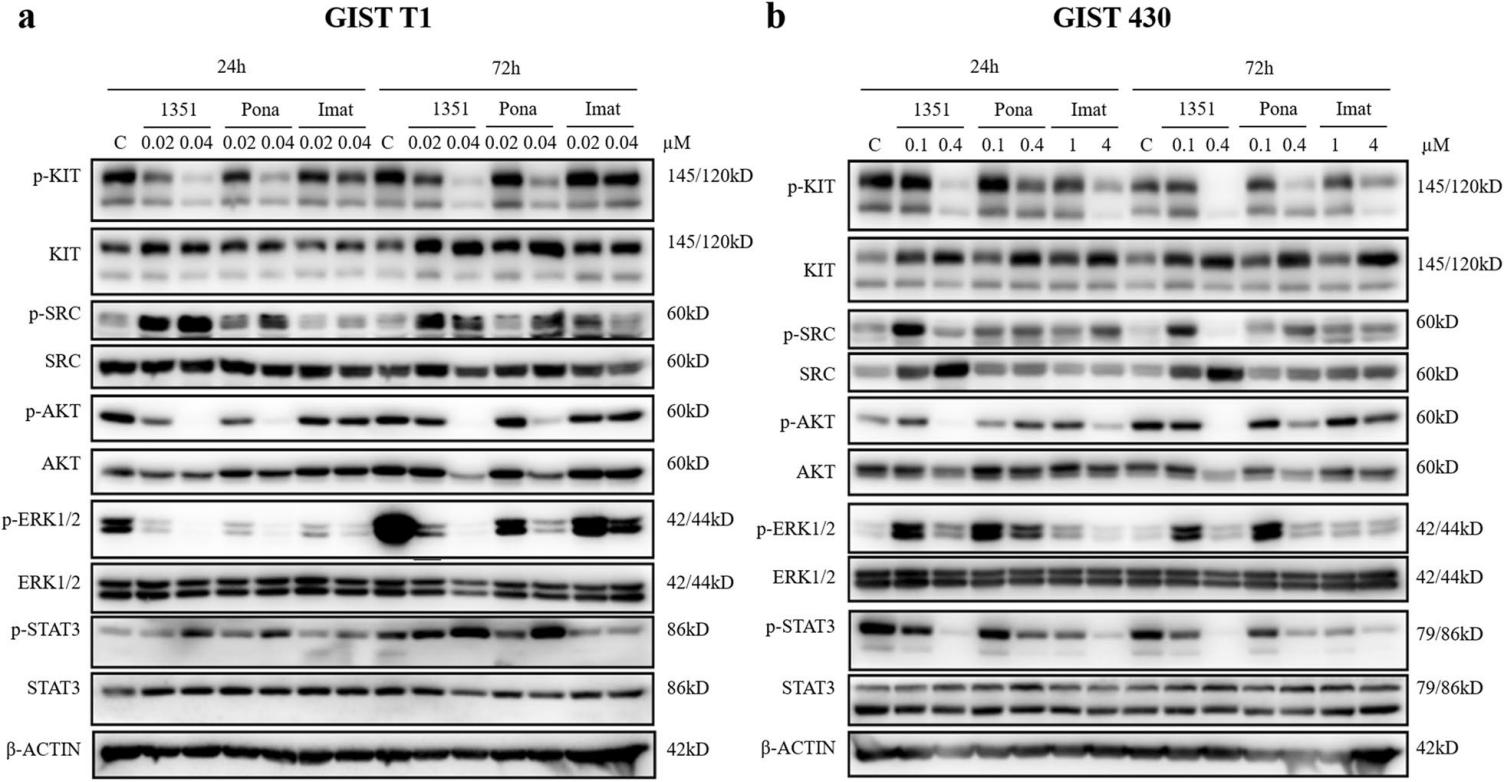
HQP1351 regulates KIT oncogenic signaling proteins in vitro. Effect of HQP1351 (1351), ponatinib (pona) and imatinib (imat) on KIT and its downstream signaling proteins in GIST T1 (**a**) and GIST 430 (**b**) cells. After the cells were treated with the indicated concentrations of HQP1351, ponatinib, or imatinib for 24 and 72 h, cell lysates were collected for Western blot analysis. Note that tenfold higher concentrations of imatinib were used in imatinib-resistant cell lines GIST 430 to order to achieve the comparable results. p, phosphorylation; C, control

To further investigate the mechanism of action of HQP1351, GIST T1 and GIST 430 cells expressing different mutant KIT kinases were treated with HQP1351 in vitro. The levels of expression and phosphorylation of KIT and the downstream signaling pathway proteins were assessed by western blotting (Fig. 3).

In the imatinib-sensitive GIST T1 cells harboring primary KIT mutations, the levels of phosphorylated KIT, AKT, SRC and ERK1/2 were significantly decreased after the treatment with HQP1351 at 0.02 and/or 0.04 μM for 24 or 72 h. In general, the inhibitory effect of HQP1351 was much greater than that of imatinib and slightly greater than ponatinib at the same concentrations (Fig. 3a). In imatinib-resistant GIST 430 cells, the levels of phosphorylated KIT and its downstream components AKT and STAT3 were significantly downregulated after the treatment with HQP1351 at 0.1 and 0.4 μM for 24 or 72 h (Fig. 3b). The inhibitory effect of HQP1351 is greater than ponatinib at the same concentrations. Even under tenfold higher concentrations, imatinib exhibited the inhibitory effect weaker than HQP1351.

The results above suggest that the effect of HQP1351 is mediated by inhibition of the phosphorylation of KIT and its downstream proteins, such as p-AKT, p-ERK1/2, p-STAT3, in KIT mutant GIST cells. In addition, the inhibitory effect of HQP1351 on these phosphorylated proteins appears to be greater than that of ponatinib and imatinib.

### HQP1351 exhibits antitumor activity in GIST xenograft models in vivo

**Fig. 4:**
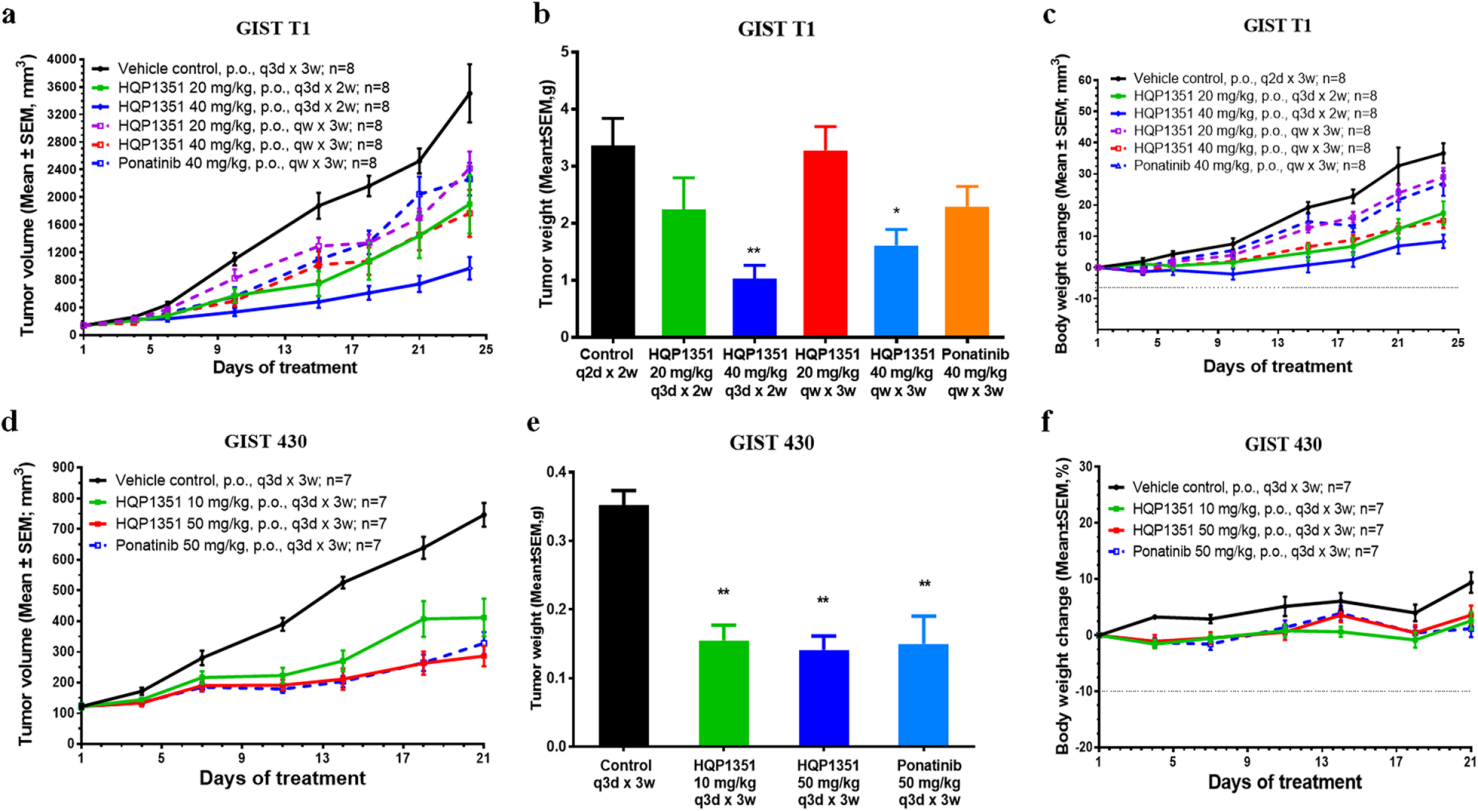
HQP1351 exhibits antitumor activity in GIST xenograft models in vivo. Growth curves of subcutaneous xenografts of GIST T1 cells (**a**), weight of subcutaneous xenografts of GIST T1 cells at the end of the experiment (**b**), and body weight of Balb/c nu/nu mice bearing GIST T1 xenograft tumors (**c**) after treatment with HQP1351 and ponatinib. Growth curves of subcutaneous xenografts of GIST 430 cells (**d**), weight of subcutaneous xenografts of GIST 430 cells at the end of the experiment (**e**), and body weight of SCID Beige mice bearing GIST 430 xenograft tumors (**f**) after treatment with HQP1351 and ponatinib. *P < 0.05 vs. control; **P < 0.01 vs. control

In vivo antitumor activity of HQP1351 in mouse xenograft models derived from GIST cell lines was evaluated. Animals bearing GIST T1 xenografts were orally administrated with HQP1351 at a dose of 20 or 40 mg/kg with a dosing schedule of once every 3 days (q3d) for 2 weeks or once weekly (qw) for 3 weeks. After the treatment, in comparison with the vehicle control, HQP1351 showed dose-dependent antitumor activity (Fig. 4a and Table 3). Under the q3d schedule, an increase in the dose of HQP1351 from 20 mg/kg to 40 mg/kg enhanced the antitumor activity (i.e. T/C%) from 56.2% (*P* < 0.01 vs. control) to 29.1% (*P* < 0.001 vs. control) at the endpoint of the experiment (d24). Similarly, under the qw schedule, the T/C value (%) of HQP1351 at 20 and 40 mg/kg were 69.1% (*P* < 0.05 vs. control) and 54.4% (*P* < 0.01 vs. control), respectively. Of note, even though no statistically significant difference between two compound treatment groups was observed, HQP1351 (T/C, 54.4%; *P* < 0.01 vs. control) showed slightly greater antitumor activity than ponatinib (T/C, 65.0%; *P* < 0.05 vs. control) at the same dosing regimen (40 mg/kg, qw × 3w). The tumor weight of each treatment group was shown in Fig. 4b. The trends of tumor weight reduction after treatment by HQP1351 and ponatinib were generally in agreement with the results of tumor volume. Significantly reduced tumor weight was found when the mice were treated with 40 mg/kg HQP1351 at the schedule of q3d × 2w (*P* < 0.01 vs. control) and qw × 3w (*P* < 0.05 vs. control). Body weight of mice treated with HQP1351 didn’t change significantly, indicating that the treatment with HQP1351 was well tolerated at the above tested doses (Fig. 4c).

**Table 3:** Summary of antitumor activity of HQP1351 in the treatment of GIST xenografts in nude mice

Model	Drug	Schedules	Dose (mg/kg)	RTV	T/C (%)
GIST T1	Control			24.78 ± 2.51	
	HQP1351	q3d × 2w	20	13.93 ± 3.26**	56.2
			40	7.21 ± 1.05**	29.1
		qw × 3w	20	17.13 ± 2.19	69.1
			40	13.47 ± 2.17**	54.4
	Ponatinib	qw × 3w	40	16.10 ± 1.75*	65.0
GIST 430	Control	q3d × 3w		6.38 ± 0.74	
	HQP1351	q3d × 3w	10	3.58 ± 0.69**	56.1
			50	2.39 ± 0.27**	37.5
	Ponatinib	q3d × 3w	50	2.80 ± 0.42**	43.9

*RTV* relative tumor volume, calculated by dividing the absolute tumor volume at the end of experiment with the absolute tumor volume at day 1

*P < 0.05 vs. control; **P < 0.01 vs. control

In the xenograft model derived from imatinib-resistant GIST 430 cells, HQP1351 was orally administrated at a dose of 10 or 50 mg/kg once every 3 days for 3 weeks (q3d × 3w). At the end of treatment (d21), the T/C (%) values of HQP1351 at 10 and 50 mg/kg were 56.1% (*P* < 0.05 vs. control) and 37.5% (*P* < 0.01 vs. control), respectively. The results revealed that HQP1351 was efficacious in GIST 430 xenografts. The antitumor activity of HQP1351 (T/C, 37.5%; *P* < 0.01 vs. control) was slightly greater than that of ponatinib (T/C, 43.9%; *P* < 0.01 vs. control) at the same dosing regimen (50 mg/kg, q3d × 3w) regardless of a lack of statistically significant difference between two agents (Fig. 4d and Table 3). The data of the tumor weight of each treatment group was consistent with the tumor volume at the endpoint (Fig. 4e). All the three treatment groups showed significantly reduced tumor weight comparing with the vehicle control group (*P* < 0.01). Body weight of mice treated with HQP1351 didn’t change significantly, indicating that the HQP1351 was well tolerated at the tested doses (Fig. 4f).

## Discussion

HQP1351 is a new generation multikinase inhibitor primarily targeting both wild-type and mutant BCL-ABL and KIT kinases. It is currently in the clinical development for the treatment of imatinib-resistant CML. In the present study, we investigated the effects of HQP1351 on KIT kinases in attempt to develop the agent as a third-generation KIT inhibitor for the treatment of imatinib-resistant GIST patients. Considering that ponatinib is a third-generation KIT inhibitor and has shown efficacy in GIST patients in clinical trials [17], we included ponatinib for comparison in the following key experiments in order to identify the differentiations between these two agents.

Biochemical assays demonstrate that HQP1351 at 10 nM exerts potent inhibitory effect (> 90%) on both wide-type and multiple mutant KIT kinases, including primary L576P and V559D mutants. Similarly, potent inhibitory effect of HQP1351 is observed on secondary mutations in the ATP-binding pocket (V559D/T670 and V559D/V654A), with > 90% of inhibition at 100 nM. In addition, HQP1351 also inhibits A-loop mutations (A829P, D816H and D816 V) with a relatively weaker activities ranging from 50 to 80% at 100 nM. Therefore, HQP1351 could be potentially useful in overcoming the secondary KIT mutations in resistant GISTs. In GIST cell lines carrying KIT mutations, very similar antiproliferative activities were observed between HQP1351 and ponatinib with the differences of IC_50_ less than twofold in GIST T1 and GIST 430 cell lines. In imatinib-resistant GIST 430 cells, HQP1351 was 17.8 times more potent than imatinib, suggesting its potential use to overcome resistance in GISTs caused by secondary mutations in ATP-pocket. Similarly, HQP1351 was also 5.3 and 36.5 times more potent than sunitinib and regorafenib respectively in the same cell line, showing the advantage over second-generation KIT inhibitors. These cellular data indicate that HQP1351 exhibits antiproliferative activities comparable with ponatinib in GIST cells carrying primary and secondary mutations. Consistent with the potent antiproliferative activity, HQP1351 shows the strongest inhibitory effect on the clonogenic ability of imatinib-resistant GIST 430 cells, as demonstrated by forming least number and smallest size of cell colonies. The inhibitory effect of HQP1351 on the migration of GIST 430 cells is also the strongest in the wound healing assay comparing with imatinib and ponatinib. Furthermore, HQP1351 showed the potent inhibitory effect on cell invasion, which excluded the possibility that the impaired wound closure was caused by HQP1351-mediated cell growth inhibition.

Cell cycle arrest and apoptosis are two effective mechanisms involved in the induction of cell death [23]. HQP1351 mainly induces G0/G1 cycle arrest in GIST 430 cells, suggesting that cell cycle arrest contributed to the antiproliferative ability of HQP1351. Previously, the cell cycle of GIST T1 cells showed similar change when it was treated with imatinib [24]. On the other hand, HQP1351 remarkably induced the apoptosis of GIST 430 cells. Therefore, the antiproliferative effect of HQP1351 against imatinib-resistant GIST 430 cells is likely caused by both cell apoptosis and cell cycle arrest. More importantly, the ability of HQP1351 to induce cell cycle arrest and cell apoptosis is superior to ponatinib.

It has been reported that, in GIST tumors, signaling mechanisms of KIT may vary depending on the exact location and type of the oncogenic KIT mutations [25]. In agreement with the observation, we illustrated the expression and phosphorylation of KIT in GIST T1, and GIST 430 cell lines. Pharmacodynamic analyses further revealed the modulation of the oncogenic KIT signal transduction pathway by HQP1351 and the differential activities of HQP1351 in comparison with ponatinib and imatinib. As expected, HQP1351 consistently inhibited phosphorylation of KIT in both imatinib-sensitive (GIST T1) and imatinib-resistant (GIST 430) GIST cell lines. Conversely, the effect of HQP1351 on KIT downstream signaling intermediates appears to be variable. Notably, downregulation of ERK1/2 phosphorylation occurred predominantly in imatinib-sensitive cell lines (GIST T1) whereas downregulation of STAT3 and SRC phosphorylation appeared mainly in imatinib-resistant cell lines (GIST 430). Notably, upregulation of p-EKR1/2 was observed in GIST 430 cells treated with imatinib, ponatinib and HQP1351. Considering the complexity of ERK signal pathway where negative feedbacks occurred as reported [26], it was speculated that the cells might upregulate the phosphorylation of ERK1/2 in response to TKI treatment. In addition, phosphorylation is a fast and dynamic process, the status of the cells and the time point of sample collection will also affect the phosphorylation level of ERK1/2. On the other hand, it was difficult to understand the upregulated level of p-SRC upon treatment which deserved further investigation. These results suggest that the signal transduction pathway underlying the effect of HQP1351 may vary among imatinib-sensitive and imatinib-resistant cell lines. In addition, the inhibitory effects of HQP1351 on phosphorylation of KIT, ERK1/2, SRC, and STAT3 are more potent than both imatinib and ponatinib in GIST T1 and GIST 430 cell lines. Our results suggest that HQP1351 is more effective in modulating the KIT signal transduction pathway and, potentially, more efficacious than ponatinib in tumor models in vivo, as well as in clinic.

In vivo antitumor activity of HQP1351 was next evaluated. Under different dosing schedules (q2d, q3d, qw), HQP1351 consistently demonstrates dose-dependent antitumor activity in xenograft models derived from GIST T1 and GIST 430 cell lines. The results suggest that HQP1351 is highly effective in inhibiting the propagation of xenograft tumors carrying not only the primary KIT mutations, but also the secondary KIT mutations in ATP-binding pocket. Furthermore, under the same dosage regimen, the antitumor activity of HQP1351 was greater than ponatinib in both GIST tumor models despite a lack of statistically significant differences between two treatment arms. Therefore, HQP1351 may become a more effective therapy for GIST patients with either primary or secondary KIT mutations. The latter accounted for 50% of imatinib-resistance mutations [27, 28]. In addition, several dosing schedules were tested, and the results will assist in the clinical trial design.

Collectively, HQP1351 is a novel multikinase inhibitor that displays potent inhibition on both wide-type and mutated KIT kinase. HQP1351 effectively overcomes the imatinib resistance induced by secondary KIT mutations and exhibits antitumor activity in xenograft tumor models of GISTs in mice. Notably, the inhibitory effects of HQP1351 on oncogenic KIT transduction pathway, as well as its antitumor activity appear to be superior to ponatinib. In addition, the safety profile of HQP1351 might be advantageous over ponatinib for the therapeutic purpose. In the phase II PACE trial, ponatinib showed increased risk of arterial occlusive events (AOEs) with increased treatment duration, and the cumulative incidence of AOEs in chronic phase (CP)-CML patients reached 31% (serious AOEs, 26%) after a 5-year follow-up [29]. The risk of life-threatening AOEs resulted in the temporarily withdrawal of ponatinib from the US market, although it later returned with enhanced warnings of these safety concerns [18]. On the other hand, most recently, HQP1351 showed manageable side effects in the phase I study to treat chronic myelogenous leukemia (CML), while it demonstrated clinical efficacy in CML resistant to current TKI-therapies including those with T315I mutation [20]. Taken together, our results implicate for therapeutic application of HQP1351 in imatinib-resistant GIST patients and, accordingly, a Phase I clinical trial has been initiated to evaluate the safety and early efficacy of HQP1351 in China.

## Supplementary information

**Additional file 1: Table S1.** Inhibition of various kinases with %Ctrl < 35% by HQP1351 at 10 nM and 100 nM

## Data Availability

The datasets generated during and/or analyzed during the current study are available from the corresponding author on reasonable request.

## Abbreviations

GIST: gastrointestinal stromal tumors
CML: chronic myelogenous leukemia
PDGFRA: platelet-derived growth factor receptor alpha
ALL: acute lymphoblastic leukemia
RTV: relative tumor volume
AOEs: arterial occlusive events
CP: chronic phase
