# Supplementary material for: Preclinical development of HQP1351, a multikinase inhibitor targeting a broad spectrum of mutant KIT kinases, for the treatment of imatinib-resistant gastrointestinal stromal tumors
Source: Cell Biosci. 2019 Oct 26;9:88. doi: 10.1186/s13578-019-0351-6 (PMC6815454; doi:10.1186/s13578-019-0351-6)
Supplement: Supplementary file 1 — Additional file 1: Table S1. Inhibition of various kinases with %Ctrl < 35% by HQP1351 at 10 nM and 100 nM [file 13578_2019_351_MOESM1_ESM.docx]

**Additional Material**

**Table S1** Inhibition of various kinases with %Ctrl <35% by HQP1351 at 10 nM and 100 nM.

| AmbitGeneSymbol | % of control | | Inhibition rate (%) | |
| --- | --- | --- | --- | --- |
|  | 10nM | 100nM | 10nM | 100nM |
| BLK | 2.4 | 0.45 | 97.6 | 99.55 |
| BRAF(V600E) | 6.4 | 0.45 | 93.6 | 99.55 |
| CSF1R | 2.2 | 0.3 | 97.8 | 99.7 |
| CSK | 2.2 | 0.85 | 97.8 | 99.15 |
| DDR1 | 0.25 | 0.45 | 99.75 | 99.55 |
| PHA8 | 11 | 0.15 | 89 | 99.85 |
| FGFR1 | 16 | 1.6 | 84 | 98.4 |
| FLT3 | 2.4 | 0.75 | 97.6 | 99.25 |
| FLT3(K663Q) | 5 | 0.6 | 95 | 99.4 |
| FLT3(N841I) | 2.9 | 0.05 | 97.1 | 99.95 |
| HCK | 4.2 | 0.4 | 95.8 | 99.6 |
| IKK-alpha | 14 | 0.7 | 86 | 99.3 |
| IKK-beta | 10 | 0.9 | 90 | 99.1 |
| LCK | 3.2 | 0.25 | 96.8 | 99.75 |
| LOK | 0.15 | 0 | 99.85 | 100 |
| LYN | 17 | 0.95 | 83 | 99.05 |
| MAP3K3 | 10 | 1 | 90 | 99 |
| MAP4K2 | 14 | 0.55 | 86 | 99.45 |
| MEK5 | 5.2 | 1.3 | 94.8 | 98.7 |
| MUSK | 20 | 0.55 | 80 | 99.45 |
| PDGFRA | 24 | 0.65 | 76 | 99.35 |
| PDGFRB | 9.5 | 0.05 | 90.5 | 99.95 |
| PFCDPK1(P.falciparum) | 21 | 1.9 | 79 | 98.1 |
| RET | 15 | 0.1 | 85 | 99.9 |
| RET(M918T) | 9.4 | 0.2 | 90.6 | 99.8 |
| SRC | 26 | 0.75 | 74 | 99.25 |
| TAK1 | 3.8 | 1.8 | 96.2 | 98.2 |
| TAOK3 | 7.9 | 0.25 | 92.1 | 99.75 |
| TIE1 | 18 | 10 | 82 | 90 |
| TIE2 | 2.8 | 0.25 | 97.2 | 99.75 |
| TNIK | 19 | 6.6 | 81 | 93.4 |
| TNK1 | 17 | 1.3 | 83 | 98.7 |
| TRKA | 0 | 0 | 100 | 100 |
| TRKB | 14 | 11 | 86 | 89 |
| TRKC | 18 | 0.9 | 82 | 99.1 |
| YES | 30 | 1.5 | 70 | 98.5 |
| ZAK | 3.8 | 0.7 | 96.2 | 99.3 |
